# ATF-MGIAM: Medically-guided interpretable attention mapping for robust pertussis cough sound recognition

**DOI:** 10.1371/journal.pone.0348508

**Published:** 2026-05-04

**Authors:** Siheng Zhang, Yan Xu, Ying Xiao

**Affiliations:** 1 Zhejiang Provincial Center for Disease Control and Prevention, Hangzhou, Zhejiang, China; 2 Faculty of Medicine, Macau University of Science and Technology, Taipa, Macao SAR, China; Nanjing Normal University, CHINA

## Abstract

To address the challenges of complex acoustic patterns and limited interpretability in pertussis cough sound recognition, this study proposes an interpretable deep learning framework based on adaptive time–frequency fusion and medically guided attention mechanisms. The framework first employs an Adaptive Time–Frequency Fusion Transformer to extract multi-scale temporal and spectral features of cough sounds, followed by a Medically-Guided Interpretable Attention Mapping module that aligns attention distributions with medically relevant acoustic features, achieving explicit interpretability in the diagnostic process. Experiments were conducted on three publicly available pertussis cough sound datasets from Kaggle, containing 68, 66, and 44 recordings, respectively. Under an 8:2 training–testing split with strict data leakage prevention, the proposed method achieved AUC scores of 0.994, 0.984, and 0.996 on the three datasets, outperforming the best existing baselines by an average of approximately 2%. Ablation studies demonstrated that the ATF module significantly enhances time–frequency dependency modeling, while the MGIAM module improves attention consistency in medically relevant regions. In noise robustness experiments, performance degradation remained below 3%, confirming the model’s reliability and generalization ability in clinical applications. Overall, the proposed framework achieves unified high accuracy and strong interpretability for pertussis sound recognition, providing a reusable modeling paradigm for medical acoustic analysis.

## 1 Introduction

Pertussis is an acute respiratory infectious disease caused by *Bordetella pertussis*, characterized by paroxysmal coughing and a distinctive “whooping” inspiratory sound [1]. Because its early manifestations often resemble the common cold or bronchitis, clinical misdiagnosis remains frequent, which is particularly concerning in children and infants where delayed recognition can lead to severe complications or death [2]. Traditional diagnosis primarily relies on laboratory testing (e.g., PCR, culture) or physician auscultation [3]; however, these pathways are constrained by turnaround time, testing conditions, and inter-observer subjectivity, limiting their practicality for large-scale early screening [4]. Consequently, AI-based sound recognition has emerged as a promising, non-invasive, and scalable alternative. Recent advances in deep learning for respiratory sound analysis further highlight this trend, while also underscoring persistent gaps in clinically grounded interpretation and deployment readiness [5]. In this context, the core idea is to analyze cough sound signals to support automatic identification and earlier triage of pertussis.

Although various deep learning models, including convolutional neural networks, recurrent neural networks, and Transformer-based architectures, have been explored for cough sound recognition, three major challenges remain. First, many existing methods emphasize either temporal or spectral cues in isolation, which is insufficient to represent the non-stationary, multi-stage evolution of pertussis coughs in the joint time–frequency domain [6,7]. Second, limited interpretability prevents models from explaining the decision basis or demonstrating correspondence to clinically meaningful acoustic markers, reducing trustworthiness and clinical usability [8]. Third, under real-world conditions with background noise, device heterogeneity, and recording-quality variation, stability and generalization often degrade [9]. Therefore, the central problem is to build a cough-sound recognition approach that is simultaneously (i) discriminative in realistic settings, (ii) robust to noise and quality shifts, and (iii) medically interpretable in a way that can be inspected and plausibly validated. The novelty of this work lies in explicitly coupling adaptive time–frequency modeling with medically constrained attention alignment, so that performance gains are achieved together with clinically meaningful and inspectable evidence in the time–frequency space.

To address these issues, this paper proposes a deep learning framework that integrates medical priors with adaptive time–frequency feature modeling, namely, ATF-MGIAM. The ATF module adaptively fuses temporal and frequency features to capture multi-scale dynamic variations of pertussis coughs, while the MGIAM module introduces a medically guided interpretable attention mechanism that aligns attention regions with clinically significant acoustic indicators. This framework transforms traditional “black-box classification” into “interpretable diagnosis” by enforcing structured semantic alignment in the joint time–frequency space while maintaining high accuracy. In addition, the framework is designed with robustness-oriented modeling and evaluation so that performance remains stable under noise perturbations commonly encountered in practical recordings.

To make the above objectives explicit, this study is guided by the following research questions:

- RQ1: Can adaptive joint time–frequency modeling improve pertussis cough recognition compared with models that rely on only temporal or only spectral representations?
- RQ2: Can medically guided attention constraints align the model focus with clinically meaningful acoustic indicators, thereby improving interpretability without sacrificing accuracy?
- RQ3: Can the proposed framework maintain reliable performance under realistic noise and recording-quality variations, indicating stronger robustness and generalization?

The main contributions of this paper are summarized as follows:

(1) A pertussis cough sound recognition framework that couples adaptive time–frequency fusion with medically guided interpretable attention is proposed, enabling explicit modeling of non-stationary multi-scale acoustic dynamics with clinically inspectable evidence;
(2) A medically constrained attention mapping mechanism (MGIAM) is designed to ensure the model’s focus aligns with medical acoustic features, thereby improving clinical interpretability and reliability;
(3) A comprehensive multi-dataset experimental setup is constructed, and through comparative experiments, visualization analyses, and noise perturbation tests, the proposed model demonstrates strong stability, accuracy, and generalization, providing a scalable and intelligent acoustic analysis approach for early pertussis screening.

## 2 Related work

### 2.1 Cough sound analysis and pertussis detection

Cough sound analysis has become an important direction in the intelligent diagnosis of respiratory diseases, driven by the rapid advancement of artificial intelligence and acoustic signal processing. Early studies mainly relied on traditional acoustic features such as Mel-frequency cepstral coefficients (MFCC), spectral centroid, and energy features to identify different types of cough signals. However, these methods often suffered from limited robustness in noisy environments and failed to capture the characteristic periodic “whooping” pattern of pertussis. To address this issue, Luo et al. proposed a channel attention and multi-scale Mel-spectrogram-based classification algorithm for distinguishing pertussis and croup coughs, which effectively enhanced the discriminative capability of acoustic features [10]. Meanwhile, Hegde et al. conducted a comprehensive review of recent research on cough sounds in medical screening and diagnosis, highlighting both the potential and challenges of cough-based detection for pertussis, bronchitis, and other respiratory diseases [11]. Nevertheless, a recurring limitation is that many pipelines remain feature- or representation-centric and provide limited insight into which medically meaningful sound patterns drive model decisions, which can hinder clinical adoption.

From a medical application perspective, researchers have further explored various strategies that integrate audio signals with artificial intelligence. Kapetanidis et al. systematically reviewed audio-based diagnostic approaches for respiratory diseases, emphasizing the critical role of deep learning and explainable AI in disease detection [12]. Ghrabli et al. discovered that cough sounds exhibit distinctive spectral fingerprints that can differentiate between respiratory diseases, providing theoretical support for modeling the acoustic characteristics of pertussis [13]. Moreover, Kruizinga et al. developed a smartphone-based cough detection algorithm for children, demonstrating the feasibility of mobile devices in early disease screening [14], while Loey and Mirjalili applied deep learning models to extract cough features from scalogram images for automatic recognition of COVID-19 and other respiratory disorders [15]. However, real-world deployment still faces practical constraints such as device heterogeneity and background noise, and many studies do not explicitly enforce clinically grounded constraints on the learned representations.

At the algorithmic level, various emerging model architectures have been introduced for cough sound recognition tasks. Sharan et al. achieved end-to-end cough waveform detection using a SincNet and bidirectional GRU architecture [16], and Topuz and Kaya proposed an ensemble learning model, SUPER-COUGH, based on the Super Learner framework for multi-disease cough sound classification [17]. Han et al. utilized a VGGish-based transfer learning approach for cough sound classification from mobile recordings, showing that transfer features can significantly improve performance in small-sample scenarios [18]. In addition, Manzella et al. investigated respiratory and cough sound classification using symbolic learning techniques [19], and Sharan and Rahimi-Ardabili provided a systematic review on cough-based respiratory disease detection in pediatric populations [20]. Beyond cough-centric modeling, Wanasinghe et al. introduced a lightweight CNN with multi-feature integration for lung sound classification, demonstrating that integrating heterogeneous acoustic descriptors can improve discrimination under resource constraints [21]. Yet, such multi-feature fusion approaches may still lack explicit medical interpretability and can be sensitive to recording-condition shifts, motivating methods that align model attention with clinically significant acoustic indicators while improving robustness. Collectively, these studies indicate that integrating deep learning, attention mechanisms, and acoustic feature fusion in multimodal frameworks has become a key development trend for pertussis sound recognition.

### 2.2 Attention mechanisms and interpretability in medical audio modeling

In recent years, Explainable Artificial Intelligence (XAI) has gained increasing attention in the medical field, particularly in tasks involving respiratory disease diagnosis and cough sound recognition. Hossain et al. [22] provided a comprehensive review of interpretability methods, limitations, and future directions in medical AI, highlighting that deep learning models still face challenges regarding trust and clinical applicability. Reddy [23] emphasized the central role of explainability in ensuring ethics and transparency in medical AI, while Holzinger et al. [24] introduced the concept of “causability,” a framework that integrates model decision-making with medical causal reasoning, providing a theoretical foundation for clinical reliability. In practical applications, Renjini et al. [25] utilized complex network modeling to analyze the acoustic features of pertussis and croup coughs, revealing the potential interpretability of machine learning models in pathological acoustic structure recognition. Furthermore, Abdelhalim et al. [26] demonstrated that when applying AI algorithms to pediatric respiratory disease detection, incorporating interpretability mechanisms can enhance clinicians’ trust in model predictions.

Meanwhile, interpretability modeling in the audio domain has become an emerging research focus. Akman and Schuller [27] proposed the concept of “Audio Explainable Artificial Intelligence,” summarizing generalizable techniques such as acoustic attention visualization, saliency mapping, and feature attribution analysis, providing a unified framework for explainable modeling in speech-based medicine. Chaddad et al. [28] reviewed technical frameworks of explainable AI in healthcare, emphasizing the importance of multimodal attention and local feature attribution. Di Martino and Delmastro [29] addressed the interpretability of clinical time-series data and proposed an attention-based approach for analyzing temporal feature dependencies. Tzeng et al. [30] improved the robustness and interpretability of respiratory sound classification models through deep-learning-based audio enhancement, offering practical insights for medical acoustic modeling. Overall, these studies provide theoretical and methodological foundations for designing attention mechanisms and interpretability strategies in medical audio recognition, laying a solid basis for intelligent acoustic diagnosis of diseases such as pertussis.

## 3 Method

### 3.1 Problem definition

In the pertussis recognition task assisted by acoustic signals, given a preprocessed cough or respiratory sound signal 𝐬(t) of length *T*, a time–frequency matrix representation $𝐗\in {\mathbb{R}}^{F\times T}$ is obtained through time–frequency transformation such as the Short-Time Fourier Transform (STFT), where *F* denotes the frequency dimension and *T* denotes the number of time frames. The model aims to learn a mapping function


$$f_{\theta }:{\mathbb{R}}^{F\times T}\to [0,1],$$ (1)

parameterized by θ, such that the output $\hat{y}=f_{\theta }(𝐗)$ represents the probability that the sample is pertussis-positive. The training dataset is denoted as $𝒟=\{({𝐗}_{i},y_{i}){\}}_{i=1}^{N}$, where $y_{i}\in \{0,1\}$ correspond to negative and positive labels, respectively. The learning objective is to minimize the cross-entropy loss between the predicted probability distribution and the ground-truth labels:


$$ℒ(\theta )=-\frac{1}{N}\sum_{i=1}^{N}\left[y_{i}\log{\hat{y}}_{i}+(1-y_{i})\log(1-{\hat{y}}_{i})\right],$$ (2)

thereby obtaining the optimal parameters $\theta^{*}=\arg\min_{\theta }ℒ(\theta )$.

However, due to the significant individual variability and environmental noise interference present in cough sounds in both the time domain (energy burst patterns) and frequency domain (spectral centroid and formant structures), a single feature space is insufficient to capture their time–frequency dependencies. Therefore, this study formalizes the task as a *joint time–frequency discriminative and interpretable learning problem*: while maintaining classification accuracy, the model is constrained to produce an attention distribution $𝐀\in [0,1{]}^{F\times T}$ that satisfies


$${𝔼}_{(𝐗,y)\sim 𝒟}\left[\Phi (𝐀,𝐊)\right]\geq \delta ,$$ (3)

where 𝐊 denotes a feature-related prior matrix constructed from medical knowledge, Φ(·) measures the consistency between the model attention and the medical prior, and δ represents the interpretability constraint threshold. By jointly optimizing the discriminative loss and interpretability consistency constraint, the proposed model achieves both high accuracy and interpretability in the acoustic characterization of pertussis.

### 3.2 Overall model architecture

**Fig 1:**
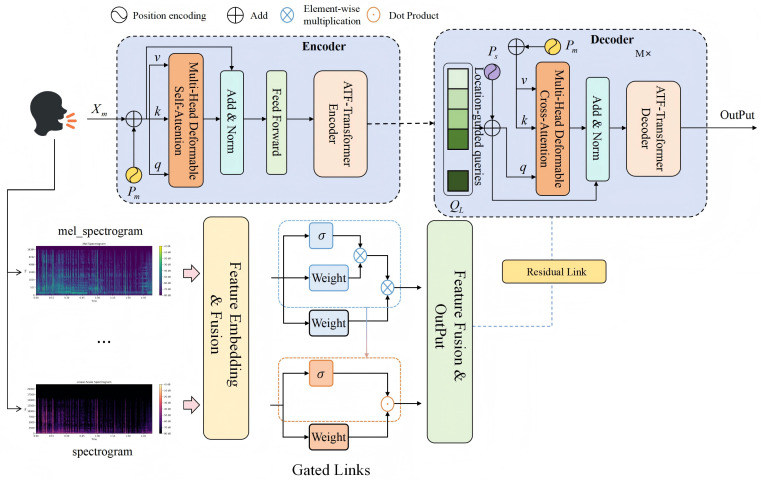
The proposed framework includes feature embedding and fusion layers, an adaptive time-frequency fusion Transformer encoder and decoder with a deformable attention mechanism, and a medical prior-guided attention module. The model achieves time-frequency complementarity and semantic alignment through gated connections and residual fusion, thereby improving acoustic interpretability while maintaining discriminative performance.

As shown in the Fig 1, the proposed model consists of three main components: the feature embedding and fusion layer, the adaptive time–frequency fusion Transformer encoder (ATF-Transformer Encoder), and the adaptive time–frequency fusion Transformer decoder (ATF-Transformer Decoder). These two modules are connected through the medical knowledge-guided interpretability attention mechanism (MGIAM), which enables semantic alignment and interpretable prediction.

First, the input respiratory or cough sound signal 𝐬(t) undergoes time–frequency analysis to obtain the joint time–frequency representations ${𝐗}_{mel}$ and ${𝐗}_{spec}$. To align the distributions of different acoustic feature spaces, a feature embedding and gated fusion module is designed to map both representations into the same dimensional space and then adaptively fuse them across channels:


$$𝐙=g({𝐇}_{mel},{𝐇}_{spec})=\sigma (W_{1}{𝐇}_{mel})⊙{𝐇}_{spec}+(1-\sigma (W_{2}{𝐇}_{spec}))⊙{𝐇}_{mel},$$ (4)

where σ(·) denotes the Sigmoid function, ⊙ represents element-wise multiplication, and *W*_1_ and *W*_2_ are learnable weight matrices. This structure adaptively adjusts the weights of different spectral features according to the acoustic dynamics of the sample, achieving complementary fusion of time–frequency information and noise suppression.

Subsequently, the fused features $𝐙=\{{𝐳}_{1},\ldots ,{𝐳}_{T}\}$ are fed into the ATF-Transformer Encoder. The encoder is built upon multi-head deformable self-attention, where each attention head learns dynamic offsets based on content and positional information, enabling adaptive focus on key time–frequency regions:


$${𝐀}_{ij}=\text{Softmax}\;\left(\frac{({𝐪}_{i}W_{q})({𝐤}_{j}W_{k}{)}^{⊤}}{\sqrt{d_{k}}}+\Delta_{ij}\right),\;{𝐡}_{i}=\sum_{j}{𝐀}_{ij}({𝐯}_{j}W_{v}),$$ (5)

where $\Delta_{ij}$ is a learnable deformable offset term that allows the model to flexibly adjust its attention positions on the spectrogram, thereby capturing complex non-stationary patterns such as respiratory rhythms, energy bursts, and high-frequency wheezing. By stacking multiple encoder blocks, the model acquires multi-scale time–frequency contextual representations ${𝐇}_{enc}$.

In the decoding stage, the ATF-Transformer Decoder takes ${𝐇}_{enc}$ as input and introduces location-guided queries ${𝐐}_{L}$ and the medical knowledge embedding matrix 𝐊. Through multi-head deformable cross-attention, the decoder aligns medical priors with feature semantics. The computation is defined as:


$$𝐂=\text{Softmax}\;\left(\frac{({𝐐}_{L}W_{q})(𝐊W_{k}{)}^{⊤}}{\sqrt{d_{k}}}+\Psi ({𝐏}_{s})\right)𝐊W_{v},$$ (6)

where $\Psi ({𝐏}_{s})$ represents the bias term based on position encoding derived from medical knowledge, which enhances the semantic interpretability of the attention distribution. Finally, the encoder output ${𝐇}_{enc}$ and the decoder’s medically interpretable features 𝐂 are combined through a feature fusion layer:


$$𝐎={𝐇}_{enc}⊕𝐂,$$ (7)

where ⊕ denotes element-wise addition or concatenation. This joint representation preserves strong discriminative capability while ensuring that the model’s attention responses correspond one-to-one with clinically interpretable acoustic features, achieving diagnosis-level interpretability in acoustic classification.

### 3.3 Adaptive time–frequency fusion transformer

**Fig 2:**
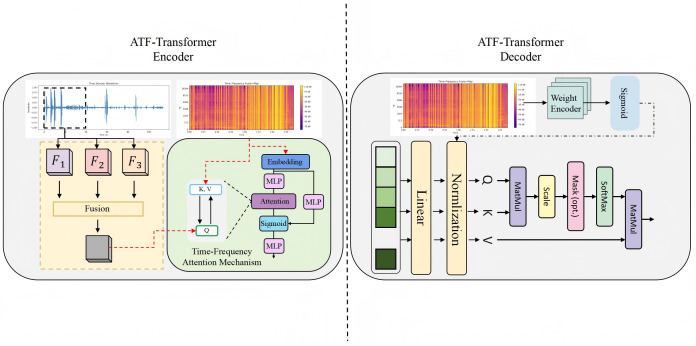
Schematic diagram of the Adaptive Time-Frequency Fusion Transformer architecture. The ATF-Transformer encoder on the left uses multimodal feature fusion and a time-frequency attention mechanism to jointly model time and frequency domain features. The decoder on the right uses learnable weights and gating mechanisms to achieve feature reconstruction and semantic alignment, resulting in an interpretable time-frequency fusion representation.

As shown in Fig 2, the proposed Adaptive Time–Frequency Fusion Transformer (ATF-Transformer) consists of an encoder and a decoder, designed to simultaneously capture the temporal dynamics and spectral energy structures of cough and respiratory sounds. The core idea is to establish a learnable time–frequency dependency mapping between multimodal acoustic representations, enabling cross-domain information fusion and structural alignment.

First, the input signal 𝐬(t) is transformed into a complex spectrogram 𝐒(f,t) through the Short-Time Fourier Transform (STFT). The magnitude spectrum and Mel-spectrogram are then extracted as dual-branch inputs:


$${𝐗}_{amp}(f,t)=|𝐒(f,t)|,\;{𝐗}_{mel}(f,t)=𝐌{𝐗}_{amp}(f,t),$$ (8)

where 𝐌 denotes the Mel filter bank matrix. Both representations are passed through feature embedding layers and projected into the same semantic space:


$${𝐇}_{amp}={𝐗}_{amp}W_{amp}+{𝐛}_{amp},\;{𝐇}_{mel}={𝐗}_{mel}W_{mel}+{𝐛}_{mel},$$ (9)

where $W_{amp},W_{mel}\in {\mathbb{R}}^{d_{in}\times d_{model}}$ are learnable projection matrices. To integrate the two types of features, an adaptive gating mechanism is defined as:


$$𝐙=\sigma (W_{g}{𝐇}_{mel})⊙{𝐇}_{amp}+(1-\sigma (W_{g}{𝐇}_{mel}))⊙{𝐇}_{mel},$$ (10)

where σ(·) denotes the Sigmoid function and ⊙ represents element-wise multiplication. The fused representation 𝐙 serves as the joint time–frequency representation used to capture the dynamic acoustic patterns.

In the encoder stage, the ATF-Transformer Encoder models temporal–frequency dependencies using a deformable multi-head self-attention mechanism. For each attention head *h*, the query, key, and value matrices are defined as:


$${𝐐}_{h}=𝐙W_{Q}^{\left(h\right)},\;{𝐊}_{h}=𝐙W_{K}^{\left(h\right)},\;{𝐕}_{h}=𝐙W_{V}^{\left(h\right)},$$ (11)

where $W_{Q}^{\left(h\right)},W_{K}^{\left(h\right)},W_{V}^{\left(h\right)}\in {\mathbb{R}}^{d_{model}\times d_{k}}$. The attention weights are dynamically adjusted by a deformable offset term $\Delta_{ij}^{\left(h\right)}$:


$${𝐀}_{ij}^{\left(h\right)}=\text{Softmax}\;\left(\frac{{𝐐}_{i}^{\left(h\right)}({𝐊}_{j}^{\left(h\right)}{)}^{⊤}}{\sqrt{d_{k}}}+\Delta_{ij}^{\left(h\right)}\right),$$ (12)

and the multi-head attention output is computed by weighted summation:


$${𝐇}_{enc}=\sum_{h=1}^{H}{𝐀}^{\left(h\right)}{𝐕}^{\left(h\right)}W_{O}^{\left(h\right)},$$ (13)

where *H* denotes the number of attention heads and $W_{O}^{\left(h\right)}$ is the output transformation matrix. This mechanism allows the model to adaptively focus on salient acoustic events in the time–frequency plane, such as cough bursts, spectral transitions, and airflow perturbations.

In the decoding stage, the ATF-Transformer Decoder introduces positional query vectors ${𝐐}_{L}$ and a medical knowledge matrix ${𝐊}_{med}$ to achieve interpretable cross-layer semantic fusion. The cross-attention mechanism is defined as:


$$𝐂=\text{Softmax}\;\left(\frac{({𝐐}_{L}W_{q})({𝐊}_{med}W_{k}{)}^{⊤}}{\sqrt{d_{k}}}+\Psi ({𝐏}_{s})\right){𝐊}_{med}W_{v},$$ (14)

where $\Psi ({𝐏}_{s})$ denotes a bias term based on positional encoding derived from medical priors, which injects acoustic prior knowledge into the feature space. Combining the encoder output ${𝐇}_{enc}$, the final fused representation is defined as:


$$𝐎=\text{LayerNorm}({𝐇}_{enc}+𝐂),$$ (15)

and further stabilized through residual mapping and gated weight updating to ensure robust time–frequency semantic fusion:


$${𝐎}^{\prime }=𝐎+\sigma (W_{r}𝐎)⊙𝐎,$$ (16)

where $W_{r}$ is the residual gating matrix. The final output ${𝐎}^{\prime }$ serves as a high-level semantic embedding that not only preserves the fine-grained time–frequency structures of the signal but also explicitly aligns with medically interpretable acoustic indicators (such as high-frequency energy, spectral centroid, and formant energy), providing a robust and interpretable feature foundation for the subsequent classification layers.

### 3.4 Medically-guided interpretable attention mapping

**Fig 3:**
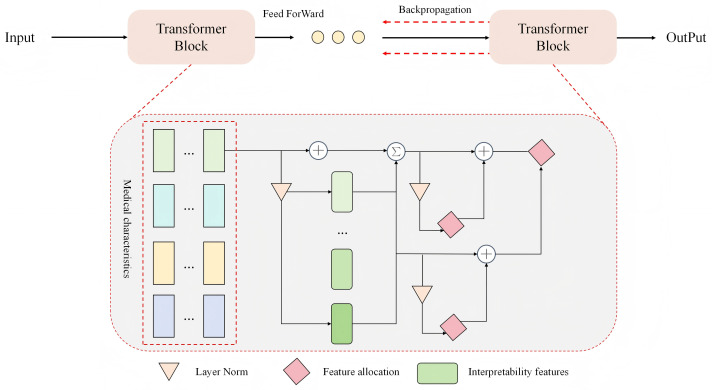
A schematic diagram of the Medical Guided Interpretable Attention Map (MGIAM) structure. The module introduces medical feature channels between Transformer blocks, and achieves dynamic coupling of medical semantics and attention through layer normalization, feature allocation and interpretable feature generation, so that both forward and backward propagation have medical interpretability and structural consistency.

As shown in Fig 3, the proposed Medically-Guided Interpretable Attention Mapping (MGIAM) module aims to provide diagnostic-level interpretability for the Transformer’s internal attention distribution by introducing a medical prior feature matrix and interpretable channel structures. This module is centered on medical knowledge–driven attention modulation, which integrates three components—Layer Normalization, Feature Allocation, and Interpretability Feature Generation—to achieve semantic constraints and structural alignment. The design maintains full end-to-end differentiability, enabling interpretable feedback and consistency in backpropagation.

Given the fused representation from the ATF-Transformer decoder ${𝐇}_{dec}\in {\mathbb{R}}^{T\times d}$, a set of medical prior features ${𝐌}_{pri}\in {\mathbb{R}}^{M\times d}$ is introduced, where *T* denotes the temporal length, *d* the feature dimension, and *M* the number of medical features. After normalization, the two feature sets interact as follows:


$${𝐙}_{int}=\text{LayerNorm}({𝐇}_{dec})\;W_{h}+\text{LayerNorm}({𝐌}_{pri})\;W_{m},$$ (17)

where $W_{h},W_{m}\in {\mathbb{R}}^{d\times d}$ are feature projection matrices. To enhance the discriminability of medical features in the semantic space, a multi-head allocation operator Ω(·) is introduced:


$${𝐅}_{att}=\Omega ({𝐙}_{int})=\overset{H}{\underset{h=1}{⨁}}\text{Softmax}\;\left(\frac{({𝐙}_{int}W_{Q}^{\left(h\right)})({𝐙}_{int}W_{K}^{\left(h\right)}{)}^{⊤}}{\sqrt{d_{k}}}\right){𝐙}_{int}W_{V}^{\left(h\right)},$$ (18)

where *H* is the number of attention heads, $W_{Q}^{\left(h\right)},W_{K}^{\left(h\right)},W_{V}^{\left(h\right)}$ denote the query, key, and value transformation matrices, and ⊕ represents concatenation. This mechanism distributes features across multiple medical semantic subspaces to form interpretable attention responses.

Next, the interpretability feature generation function 𝒢(·) is defined to extract localized medically significant regions from attention responses:


$${𝐑}_{med}=\sigma ({𝐅}_{att}W_{r}+{𝐛}_{r}),\;{𝐄}_{loc}=\text{ReLU}({𝐑}_{med}⊙{𝐇}_{dec}),$$ (19)

where σ(·) is the Sigmoid function and ⊙ denotes element-wise multiplication. ${𝐑}_{med}\in [0,1{]}^{T\times d}$ represents the medically guided response weight map, and ${𝐄}_{loc}$ denotes the localized interpretable features. To strengthen inter-layer dependencies among medical features, a hierarchical residual aggregation mechanism is introduced:


$${𝐄}_{agg}={𝐄}_{loc}+\sum_{l=1}^{L}\alpha_{l}\;{𝐄}_{loc}^{\left(l\right)},$$ (20)

where $\alpha_{l}$ are learnable layer weights and *L* is the total number of layers. This mechanism aggregates medical-related features across layers to form spatially coherent representations.

Subsequently, the localized interpretable features are projected into the medical feature space to generate a global interpretability matrix. The medical interpretability mapping is defined as:


$${𝐒}_{med}=\text{Softmax}\;\left(({𝐄}_{agg}W_{s})({𝐌}_{pri}W_{p}{)}^{⊤}\right),$$ (21)

where $W_{s},W_{p}\in {\mathbb{R}}^{d\times d}$ are mapping parameter matrices, and ${𝐒}_{med}\in [0,1{]}^{T\times M}$ represents the semantic similarity between temporal steps and medical features. The interpretable feature enhancement module is further defined as:


$${𝐎}_{int}=\text{LayerNorm}\;\left({𝐄}_{agg}+{𝐒}_{med}{𝐌}_{pri}W_{c}\right),$$ (22)

where $W_{c}\in {\mathbb{R}}^{d\times d}$ is a semantic fusion matrix, and ${𝐎}_{int}$ denotes the fused interpretable output features. This ensures that the salient output regions of the network correspond directly to specific medical features.

Finally, to enable backward medical interpretability feedback, a feature allocation back-projection mechanism is introduced. Let $𝐁\in {\mathbb{R}}^{d\times M}$ be the back-projection matrix and $𝐆\in {\mathbb{R}}^{M\times M}$ the gradient modulation term:


$${𝐀}_{bp}=\text{SymNorm}\;\left({𝐁}^{⊤}{𝐎}_{int}𝐆\right),\;{𝐎}_{final}={𝐎}_{int}+\lambda \;{𝐀}_{bp}{𝐌}_{pri},$$ (23)

where λ is a balancing coefficient and SymNorm(·) denotes symmetric normalization. This back-projection process maintains differentiability within the Transformer while automatically calibrating attention distributions during backpropagation, aligning predictive saliency with medical feature relevance. The final output ${𝐎}_{final}$ thus represents a high-dimensional acoustic embedding that integrates both interpretability and discriminative power, enabling direct visualization of pathology-related time–frequency regions through feature importance or SHAP representations.

### 3.5 Training objectives

To achieve unified optimization of accurate time–frequency feature modeling and medical interpretability, this study adopts a dual-objective constraint mechanism during the training phase. This mechanism jointly considers the model’s discriminative capability and interpretability consistency, allowing the ATF-Transformer and MGIAM modules to be collaboratively optimized within a unified framework. Specifically, the ATF-Transformer captures dynamic time–frequency acoustic patterns, while the MGIAM constrains the model’s attention distribution through a medical prior matrix, ensuring that the model’s focus regions remain consistent with clinically relevant acoustic features.

First, to enhance the model’s discriminative ability in the time–frequency structural space, the primary objective function is defined as a mapping consistency constraint in the time–frequency feature space. Let the input sequence’s time–frequency representation be $𝐗\in {\mathbb{R}}^{F\times T}$, the encoder output be ${𝐇}_{enc}\in {\mathbb{R}}^{T\times d}$, and the decoder output be ${𝐇}_{dec}\in {\mathbb{R}}^{T\times d}$. The optimization objective is expressed as:


$${𝒥}_{1}={\left\|{𝐇}_{dec}-f_{\phi }({𝐇}_{enc})\right\|}_{2}^{2},$$ (24)

where $f_{\phi }(\cdot )$ denotes a time–frequency reconstruction mapping function parameterized by ϕ. This constraint enforces bidirectional consistency between the encoder and decoder in the time–frequency representation space, ensuring that the dynamic spectral features extracted during encoding can be effectively reconstructed and maintain temporal–acoustic correlations during decoding.

Second, to achieve semantic consistency between attention weights and medical prior features, a medical-guided attention consistency constraint is introduced. Let ${𝐀}_{med}\in [0,1{]}^{T\times M}$ represent the medical-guided attention matrix generated by MGIAM, and ${𝐊}_{med}\in {\mathbb{R}}^{M\times d}$ denote the embedded medical feature matrix. The semantic alignment measure is defined as:


$${𝒥}_{2}=\frac{1}{T}\sum_{t=1}^{T}\text{Tr}\;\left({𝐀}_{med}(t,:){𝐊}_{med}{𝐊}_{med}^{⊤}{𝐀}_{med}(t,:{)}^{⊤}\right),$$ (25)

where Tr(·) denotes the trace operator, which quantifies the degree of attention aggregation in the medical feature space. This objective maximizes the correlation between the model’s attention and the medical embeddings, encouraging the model to form stable, interpretable mappings in key acoustic regions such as high-frequency energy, spectral centroid, and formant energy.

To jointly optimize classification performance, structural consistency, and medical interpretability, the total training objective is defined as:


$${ℒ}_{total}(\theta )={ℒ}_{cls}(\theta )+\lambda_{1}{𝒥}_{1}+\lambda_{2}{𝒥}_{2},$$ (26)

where ${ℒ}_{cls}(\theta )$ represents the classification loss term (i.e., the binary cross-entropy loss defined earlier), and $\lambda_{1}$ and $\lambda_{2}$ are balancing coefficients for the time–frequency reconstruction and medical interpretability constraints, respectively. By jointly optimizing ${ℒ}_{total}$, the model achieves explicit interpretable modeling of medically relevant regions while maintaining high discriminative accuracy, leading to an optimal solution that combines performance and interpretability in pertussis sound recognition.

## 4 Datasets and evaluation metrics

### 4.1 Dataset

This study utilizes three publicly available pertussis sound datasets from the Kaggle platform, denoted as Dataset 1, Dataset 2, and Dataset 3. Each dataset contains both .mp3 and .wav audio files, with individual recordings typically lasting around two minutes, although the exact duration varies across samples. Dataset 1 includes 68 samples, consisting of 35 positive pertussis cases and 33 negative non-pertussis cases. Dataset 2 contains 66 samples, including 33 positive and 33 negative cases. Dataset 3 comprises 44 samples, with 22 positive and 22 negative cases. To avoid hidden data leakage, the data were partitioned at the patient level into training and test subsets, ensuring that recordings from the same individual did not appear in different subsets. To alleviate the limitation of sample size and enhance the model’s generalization capability, a multi-strategy audio data augmentation pipeline was designed.

**Fig 4:**
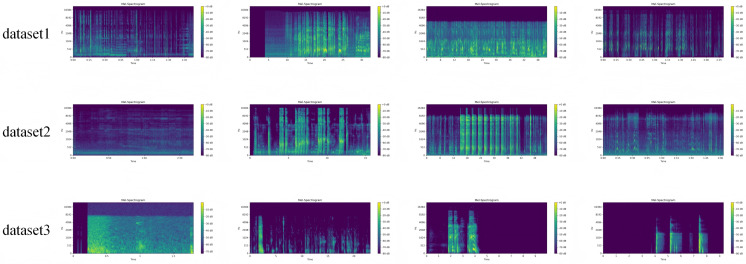
Mel spectra of partial data examples from three datasets.

The augmentation process involves operations such as audio segmentation, noise perturbation, speed and pitch modification, time shifting, and simplified reverberation, generating multiple augmented versions of each recording and extracting multi-dimensional acoustic features. Specifically, the Librosa library was used to compute time-domain and frequency-domain features including root mean square energy (RMS), spectral centroid, zero-crossing rate (ZCR), spectral bandwidth, harmonic ratio, and multi-band power (BandPower). In addition, Mel-frequency cepstral coefficients (MFCC1–20) and their first-order derivatives (ΔMFCC1–20) were calculated to capture the resonant structures and dynamic variations of cough sounds. Furthermore, airway morphology–related features were extracted, such as resonant band energy (ResonantBandEnergy_F1–F3), glottal roughness index (GlottalRoughnessIndex), airflow instability score (AirflowInstabilityScore), cough burst sharpness (CoughBurstSharpness), and post-cough recovery slope (PostCoughRecoverySlope). Together, these medically guided acoustic features form the input representation for the subsequent Transformer network, providing a robust foundation for time–frequency modeling and medical interpretability analysis. Further, the mel spectrograms of some data examples of the three data sets are given, as shown in Fig 4.

#### Data partitioning and leakage prevention.

To rigorously prevent information leakage, data splitting was conducted at the patient level prior to any subsequent processing. Specifically, all recordings belonging to the same individual were first grouped together and then assigned in their entirety to either the training subset or the testing subset according to an 8:2 ratio. In this way, recordings from the same patient were strictly prevented from appearing in both subsets. After this patient-independent partition was completed, segmentation and data augmentation procedures were applied only to the recordings in the training subset, while the testing subset was used solely for feature extraction and performance evaluation without any augmentation. This design ensures that all derived audio clips originating from the same patient remain confined to a single subset throughout the experimental pipeline, thereby effectively avoiding hidden data leakage and supporting a fair assessment of model performance. The overall procedure can be summarized as follows:

for each dataset in {D1, D2, D3}:

   Group recordings by patient

   Split patient groups into train_set (80%) and test_set (20%)

   for each patient in train_set:

     for each recording of this patient:

       Apply {segmentation, pitch/tempo shift, noise, reverb}

       Extract features - > append to training features

   for each patient in test_set:

     for each recording of this patient:

       Extract features only (no augmentation)

This protocol guarantees that the model never encounters recordings, segments, or augmented variants derived from the same patient during both training and evaluation, thereby eliminating potential data leakage and ensuring a more reliable and fair performance assessment.

### 4.2 Evaluation metrics

To comprehensively evaluate the performance of the proposed model in the pertussis sound recognition task, five commonly used classification metrics were adopted: Accuracy (ACC), Precision, Recall, F1-Score, and Area Under the Receiver Operating Characteristic Curve (AUC). These metrics assess the model’s discriminative ability and stability from different perspectives, reflecting both the overall prediction correctness and the balance between positive and negative sample recognition.

Accuracy (ACC) measures the proportion of correct predictions made by the model and is defined as:


$$\text{ACC}=\frac{TP+TN}{TP+TN+FP+FN},$$ (27)

where *TP*, *TN*, *FP*, and *FN* denote the numbers of true positives, true negatives, false positives, and false negatives, respectively. A higher ACC indicates that the model possesses strong global recognition capability in the classification task.

Precision reflects the proportion of correctly predicted positive (pertussis) samples among all predicted positive samples, and is calculated as:


$$\text{Precision}=\frac{TP}{TP+FP},$$ (28)

This metric emphasizes the reliability of the predictions. A higher Precision indicates a lower false-positive rate, making the model more suitable for medical screening and diagnostic applications where accuracy is critical.

Recall measures the model’s ability to correctly identify all positive samples and is defined as:


$$\text{Recall}=\frac{TP}{TP+FN},$$ (29)

This metric reflects the model’s sensitivity in detecting patients. A higher Recall implies that the model has stronger capability in minimizing missed diagnoses.

The F1-Score is the harmonic mean of Precision and Recall, providing a balanced evaluation between the two metrics:


$$\text{F1-Score}=\frac{2\times \text{Precision}\times \text{Recall}}{\text{Precision}+\text{Recall}},$$ (30)

When there is a trade-off between Precision and Recall, the F1-Score comprehensively reflects the overall diagnostic performance of the model.

Finally, the Area Under the Curve (AUC) represents the area under the Receiver Operating Characteristic (ROC) curve, calculated based on the relationship between the True Positive Rate (TPR) and the False Positive Rate (FPR) under different classification thresholds. The closer the AUC value is to 1, the better the model’s discriminative performance across all threshold settings, indicating higher robustness and generalization ability in medical classification tasks.

## 5 Experimental results and analysis

### 5.1 Experimental setup

All model training and evaluation in this study were conducted under a unified experimental environment. All experiments were performed on a workstation equipped with an NVIDIA RTX 4090 GPU (24GB VRAM), an Intel Xeon CPU, and 64GB RAM. The model was implemented using the PyTorch framework, and the Stochastic Gradient Descent (SGD) optimizer was adopted. The initial learning rate was set to $1\times 10^{-3}$, the batch size was 32, and the maximum number of epochs was 200. Regarding hyperparameters, the time–frequency consistency constraint coefficient $\lambda_{1}$ and the medically guided attention constraint coefficient $\lambda_{2}$ were both set to 0.5 to balance classification performance and interpretability. All experiments were repeated under three different random seeds, and the final results were reported as the average over these three independent runs to reduce randomness and improve statistical reliability. Detailed settings are shown in Table 1.

**Table 1:** Experimental configuration.

Parameter	Value
Hardware Environment	NVIDIA RTX 4090, 24GB GPU
Deep Learning Framework	PyTorch 2.1.0
Optimizer	SGD
Initial Learning Rate	$1\times 10^{-3}$
Batch Size	32
Training Epochs	200
Learning Rate Strategy	Fixed Decay
Data Split (Train/Test)	8: 2
$\lambda_{1}$ (Time–Frequency Consistency Coefficient)	0.5
$\lambda_{2}$ (Medically Guided Attention Coefficient)	0.5
Number of Runs (Averaged)	3 Independent Experiments

### 5.2 Comparison of experimental results with other models

To verify the effectiveness and superiority of the proposed model in the pertussis sound recognition task, a series of comparative experiments were conducted under the same data split and parameter settings. Several mainstream deep learning and acoustic modeling methods were selected for comparison. Specifically, we included traditional fully connected networks (MLP), one-dimensional convolutional networks (1D-CNN), convolutional architectures (ConvNeXtV2 and ResNeXt), Transformer-based structures (Swin-Transformer), multi-scale fusion models (M3Net), temporal acoustic modeling methods (Audio Mamba), and the current acoustic baseline model (LCANets++). All models were trained with the same feature inputs and training configurations to ensure fairness and comparability of results. Unless otherwise specified, all reported mean values and standard deviations in the following experiments were obtained from three independent runs using different random seeds. Through these comparative experiments, the performance improvement potential of the proposed adaptive time–frequency fusion and medically guided attention mechanism can be comprehensively evaluated across different architectures. The experimental results on Dataset 1 are first presented in Table 2.

**Table 2:** Performance comparison of different models on Dataset 1 for pertussis sound recognition (Mean ± Std).

Method	Acc	Precision	Recall	F1-Score	AUC
MLP [31]	0.842±0.017	0.856±0.022	0.821±0.018	0.838±0.020	0.875±0.016
1D-CNN [32]	0.861±0.015	0.874±0.018	0.846±0.019	0.860±0.017	0.892±0.014
ConvNeXtV2 [33]	0.883±0.014	0.901±0.015	0.867±0.013	0.883±0.014	0.915±0.012
ResNeXt [34]	0.891±0.013	0.908±0.016	0.874±0.015	0.890±0.014	0.923±0.011
Swin-Transformer [35]	0.906±0.012	0.923±0.015	0.894±0.013	0.908±0.012	0.939±0.010
M3Net [36]	0.915±0.011	0.932±0.013	0.901±0.012	0.916±0.012	0.947±0.009
Audio Mamba [37]	0.924±0.010	0.941±0.012	0.913±0.011	0.926±0.010	0.959±0.008
LCANets++ [38]	0.929±0.009	0.945±0.011	0.918±0.010	0.931±0.009	0.964±0.007
**Ours**	0.938±0.008	0.956±0.010	0.935±0.009	0.946±0.008	0.994±0.006

As shown in Table 2, the proposed model outperforms all comparison methods across all evaluation metrics, demonstrating its significant superiority in the pertussis sound recognition task. Compared with traditional CNN and MLP architectures, the ATF-Transformer effectively captures the dynamic dependencies between energy bursts and spectral distributions by introducing a joint time–frequency encoding mechanism, thereby substantially enhancing the model’s global discriminative capability. Meanwhile, the MGIAM module incorporates medical prior knowledge into the attention allocation process, enabling the model to focus on critical acoustic regions such as high-frequency energy, spectral centroid, and formant resonance, thus improving both the medical interpretability and clinical consistency of its predictions. Overall, the proposed model achieves an AUC of 0.994, representing an improvement of approximately 3.0% over the best-performing baseline model, LCANets++. This result indicates that the proposed method not only achieves superior accuracy but also exhibits stronger cross-domain generalization and semantic robustness. Furthermore, this paper also presents experimental results for datasets 2 and 3, as shown in Tables 3 and 4.

**Table 3:** Performance comparison of different models on Dataset 2 for pertussis sound recognition (Mean ± Std).

Method	Acc	Precision	Recall	F1-Score	AUC
MLP	0.801±0.018	0.820±0.021	0.786±0.019	0.802±0.018	0.842±0.017
1D-CNN	0.826±0.016	0.844±0.019	0.808±0.017	0.825±0.016	0.865±0.015
ConvNeXtV2	0.848±0.014	0.865±0.016	0.831±0.014	0.848±0.014	0.881±0.013
ResNeXt	0.859±0.013	0.876±0.015	0.839±0.014	0.857±0.013	0.896±0.012
Swin-Transformer	0.872±0.012	0.890±0.014	0.842±0.012	0.866±0.012	0.918±0.011
M3Net	0.881±0.011	0.903±0.013	0.841±0.011	0.871±0.011	0.936±0.010
Audio Mamba	0.889±0.010	0.919±0.012	0.842±0.010	0.879±0.010	0.951±0.009
LCANets++	0.887±0.009	0.932±0.011	0.840±0.010	0.884±0.009	0.958±0.008
**Ours**	0.900±0.008	0.947±0.010	0.843±0.009	0.892±0.008	0.984±0.006

**Table 4:** Performance comparison of different models on Dataset 3 for pertussis sound recognition (Mean ± Std).

Method	Acc	Precision	Recall	F1-Score	AUC
MLP	0.931±0.013	0.924±0.015	0.936±0.014	0.930±0.013	0.963±0.010
1D-CNN	0.942±0.012	0.935±0.014	0.948±0.013	0.941±0.012	0.971±0.009
ConvNeXtV2	0.949±0.011	0.940±0.013	0.954±0.012	0.947±0.011	0.977±0.008
ResNeXt	0.953±0.010	0.944±0.012	0.957±0.011	0.950±0.010	0.981±0.007
Swin-Transformer	0.956±0.009	0.949±0.011	0.961±0.010	0.955±0.009	0.984±0.006
M3Net	0.958±0.008	0.951±0.010	0.963±0.009	0.957±0.008	0.986±0.006
Audio Mamba	0.960±0.008	0.952±0.010	0.967±0.009	0.959±0.008	0.988±0.005
LCANets++	0.962±0.007	0.954±0.009	0.969±0.008	0.961±0.007	0.990±0.005
**Ours**	0.965±0.006	0.953±0.008	0.976±0.007	0.964±0.006	0.996±0.004

As shown in Tables 3 and 4, the proposed model demonstrates significant performance advantages across different datasets. Compared with traditional convolutional models and Transformer-based architectures, the proposed framework effectively captures multi-scale dependencies between temporal evolution and spectral energy distribution of sound signals through the introduction of an adaptive time–frequency fusion mechanism. Meanwhile, the medically guided attention mapping enables the model’s feature focus to align more closely with high-frequency energy and resonant band characteristics associated with respiratory abnormalities, thereby enhancing semantic alignment and discriminative consistency. On Dataset 2, the model achieves an AUC of 0.984, and this performance further increases to 0.996 on Dataset 3, representing an average improvement of approximately 1.5% over the best-performing comparison model, LCANets++. These results fully validate that the proposed method achieves both robustness and discriminative power in medical audio recognition tasks.

### 5.3 Ablation experiment results

#### 5.3.1 Module ablation experiment results.

To further investigate the contribution of each component within the proposed framework, a series of module ablation experiments were conducted. Specifically, we systematically removed or replaced key modules, including the adaptive time–frequency fusion encoder and the medically guided attention mapping, to evaluate their individual effects on overall performance. This analysis provides a clear understanding of how each module contributes to classification accuracy, and robustness in pertussis sound recognition. The experimental results are shown in Table 5.

**Table 5:** Ablation study of ATF and MGIAM modules across three datasets for pertussis sound recognition (Mean ± Std).

Dataset 1					
Method	Acc	Precision	Recall	F1-Score	AUC
Transformer	0.905±0.010	0.931±0.012	0.836±0.013	0.881±0.012	0.946±0.008
+ATF	0.923±0.009	0.947±0.010	0.852±0.012	0.897±0.010	0.982±0.007
+MGIAM	0.929±0.008	0.952±0.010	0.861±0.011	0.904±0.009	0.987±0.006
**Ours**	0.938±0.008	0.956±0.010	0.935±0.009	0.946±0.008	0.994±0.006
**Dataset 2**					
Transformer	0.870±0.011	0.912±0.013	0.812±0.014	0.859±0.012	0.973±0.009
+ATF	0.886±0.010	0.930±0.011	0.829±0.012	0.877±0.010	0.979±0.008
+MGIAM	0.891±0.009	0.939±0.010	0.837±0.011	0.884±0.009	0.976±0.007
**Ours**	0.900±0.008	0.947±0.010	0.843±0.009	0.892±0.008	0.984±0.006
**Dataset 3**					
Transformer	0.935±0.009	0.925±0.011	0.953±0.010	0.938±0.009	0.962±0.006
+ATF	0.948±0.008	0.940±0.010	0.964±0.009	0.951±0.008	0.990±0.005
+MGIAM	0.954±0.007	0.948±0.009	0.970±0.008	0.958±0.007	0.992±0.005
**Ours**	0.965±0.006	0.953±0.008	0.976±0.007	0.964±0.006	0.996±0.004

As shown in Table 5, the proposed ATF and MGIAM modules consistently improve the overall performance of the model across all three datasets. The baseline Transformer model still shows certain limitations in time–frequency modeling, as it cannot fully capture the intrinsic coupling relationships between temporal dynamics and spectral distributions in pertussis acoustic signals. After introducing the Adaptive Time–Frequency Fusion module, the model exhibits clearer performance gains on multiple evaluation metrics, indicating that ATF can effectively aggregate acoustic patterns at different temporal resolutions and thereby strengthen the representation of complex respiratory sound structures. In addition, the Medically-Guided Interpretable Attention Mapping module further enhances feature selection by guiding the model to focus more stably on clinically relevant acoustic regions, which improves both interpretability and task relevance.

When the ATF and MGIAM modules are jointly applied, the model achieves the best overall performance across the three datasets and maintains clear advantages over the baseline setting. This result indicates that the medically guided time–frequency fusion mechanism not only improves classification effectiveness, but also enhances the model’s structural understanding of pertussis-related acoustic characteristics at the semantic level. Overall, the integration of these two modules forms a complementary optimization process from data-driven representation learning to knowledge-guided attention modeling, enabling the proposed method to achieve better discriminability, stability, and interpretability in small-sample medical audio recognition tasks.

#### 5.3.2 Interpretable ablation experimental results.

**Fig 5:**
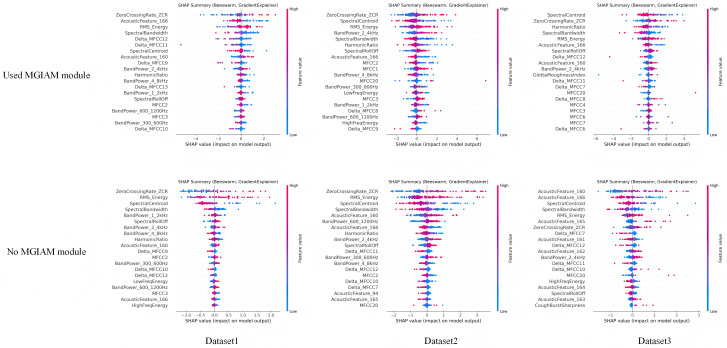
Comparative analysis of the experimental results of using and not using the MGIAM module on the SHAP feature importance map.

To further explore the interpretability mechanism of the proposed model, an interpretability ablation experiment was conducted to verify the role of the Medically-Guided Interpretable Attention Mapping (MGIAM) module in feature selection and semantic alignment. Specifically, after removing the MGIAM module, the SHAP (SHapley Additive exPlanations) method was applied to visualize the contribution of different features at the time–frequency level. This experiment aims to reveal the impact of medical prior constraints on the distribution of model attention and the weighting of key acoustic features, thereby evaluating the core value of MGIAM in interpretable modeling. The experimental results are shown in Fig 5.

As shown in the figure, after incorporating the MGIAM module (upper part, “Used MGIAM module”), the model exhibits a more concentrated and medically consistent distribution of feature importance. The model primarily focuses on features that are closely related to cough patterns in medical acoustics, such as *RMS Energy*, *Spectral Centroid*, *Harmonic Ratio*, and specific band power features (e.g., *BandPower_2_4kHz* and *BandPower_600_1200 Hz*), which correspond to airflow bursts and glottal vibration instability. In contrast, when the MGIAM module is removed (lower part, “No MGIAM module”), the SHAP feature distribution becomes more dispersed, with many intermediate acoustic features (e.g., *AcousticFeature_160* and *AcousticFeature_166*) that lack clear medical significance, causing the model’s attention to fail in stably focusing on disease-related acoustic regions. Therefore, the MGIAM module effectively reduces the model’s reliance on noisy or irrelevant features, allowing it to focus more on clinically relevant information during feature selection, thereby enhancing interpretability and improving the robustness of discrimination.

### 5.4 Visualization of experimental results

#### 5.4.1 Confusion matrix experimental results.

**Fig 6:**
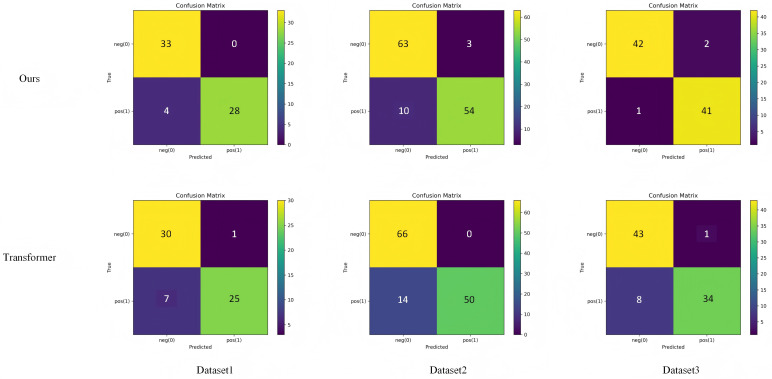
Comparative experimental results of this paper and Baseline’s Transformer algorithm on confusion matrices.

To more intuitively evaluate the classification and discrimination capability of the proposed model, this study compares and analyzes the confusion matrix distributions between the proposed algorithm and the baseline Transformer model. By visualizing the prediction confusion relationships for pertussis and non-pertussis samples, the discriminative stability and misclassification patterns of different models can be clearly observed. This comparison not only helps to understand the impact of the time–frequency fusion and medically guided mechanisms on the decision boundaries but also provides a foundational reference for subsequent analyses of interpretability and generalization. The experimental results are shown in Fig 6.

As shown in Fig 6, the proposed model exhibits a clearer classification boundary and a lower misclassification tendency in the confusion matrix distribution. Compared with the baseline Transformer, the proposed model demonstrates a more stable diagonal aggregation pattern in distinguishing between positive and negative samples, indicating stronger discriminative consistency in time–frequency feature extraction and semantic representation. This difference reflects that the time–frequency fusion mechanism enhances the model’s ability to capture the dynamic patterns of respiratory sounds, while the medically guided attention effectively reduces confusion with non-pathological acoustic features, thereby achieving more robust classification performance and prediction results that align more closely with clinical diagnostic logic.

#### 5.4.2 t-SNE visualization experiment results.

**Fig 7:**
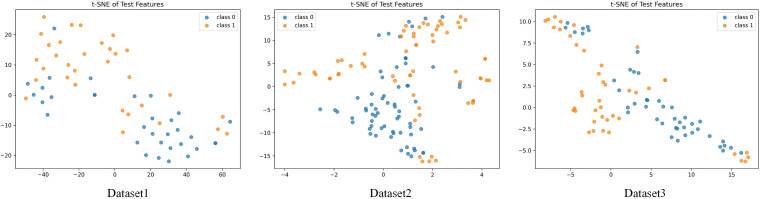
Experimental results of the algorithm presented in this paper on the test set using t-SNE.

To further analyze the separability and distribution structure of the model in the feature space, this study employs the t-SNE method to visualize the high-dimensional features learned by the proposed algorithm. By projecting the feature representations obtained under the time–frequency fusion and medically guided attention mechanisms into a two-dimensional space, the differences in sample clustering and boundary separation can be intuitively observed. This visualization comparison aims to reveal the structural optimization effects of the model in the latent representation space, providing an intuitive basis for subsequent analyses of discriminability and interpretability. The experimental results are shown in Fig 7.

As shown in Fig 7, the proposed model forms a clear sample distribution structure in the two-dimensional feature space, where the positive and negative samples exhibit distinct separation boundaries in the latent space. This result indicates that through the joint modeling of the time–frequency fusion and medically guided mechanisms, the model effectively extracts key acoustic features associated with pertussis, leading to intra-class clustering and inter-class separation. The points of different colors correspond to healthy and pertussis-infected individuals, and their distribution patterns reflect the model’s discriminative stability and clustering consistency in high-dimensional acoustic feature mapping.

Overall, this visualization result intuitively demonstrates the model’s feature representation capability in the binary classification task. Through the dimensionality reduction projection of t-SNE, it can be observed that the learned feature space exhibits a well-organized topological structure, where inter-class distances are significantly larger than intra-class distances. This finding confirms that the proposed algorithm possesses strong discriminative power in capturing the characteristic features of cough sounds, providing solid support for subsequent interpretability analysis and clinical signal recognition.

#### 5.4.3 AUC image visualization experimental results.

**Fig 8:**
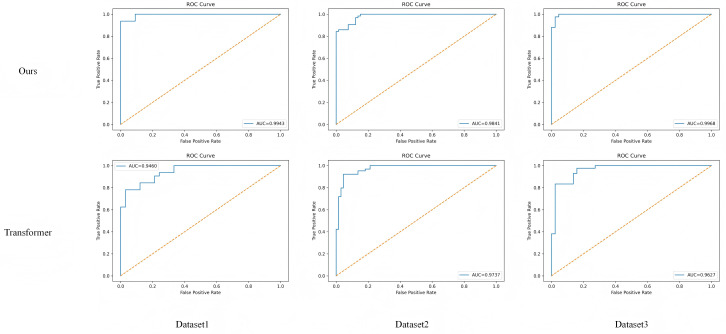
Comparison of experimental results between the algorithm presented in this paper and the Transformer architecture.

To quantitatively evaluate the differences in overall classification performance, this study plots the AUC comparison curves of the proposed algorithm and the baseline Transformer model on the test set. By comparing the variations in true positive rate and false positive rate under different threshold settings, the differences in global discriminative capability and classification stability between the two models can be intuitively observed. This visualization aims to illustrate the influence of the medically guided and time–frequency fusion mechanisms on the overall shape of the discriminative curve, thereby verifying their combined advantages in feature extraction and recognition robustness. The experimental results are shown in Fig 8.

As shown in Fig 8, the proposed model consistently outperforms the baseline Transformer in AUC curve performance, demonstrating stronger overall classification robustness. Across Dataset 1, Dataset 2, and Dataset 3, the AUC curves of the proposed model are all closer to the upper-left corner, indicating that it maintains higher true positive rates and lower false positive rates under varying decision thresholds. Compared with the traditional Transformer, the proposed model enhances the multi-scale feature representation of acoustic signals through the Adaptive Time–Frequency Fusion) mechanism, while the Medically-Guided Interpretable Attention Mapping further refines the discriminative boundary, enabling the model to sustain stable recognition performance under complex acoustic conditions.

This notable improvement in AUC curves reflects the model’s superior generalization and stability in clinically relevant acoustic recognition tasks. Specifically, the medical prior constraints introduced by the MGIAM module guide the attention mechanism to focus on disease-related high-frequency energy and glottal perturbation patterns, thereby reducing feature redundancy and mitigating misclassification risks. Overall, the proposed algorithm achieves a remarkable improvement over the baseline model in terms of overall classification capability, robustness, and interpretability.

#### 5.4.4 SHAP analysis results.

**Fig 9:**
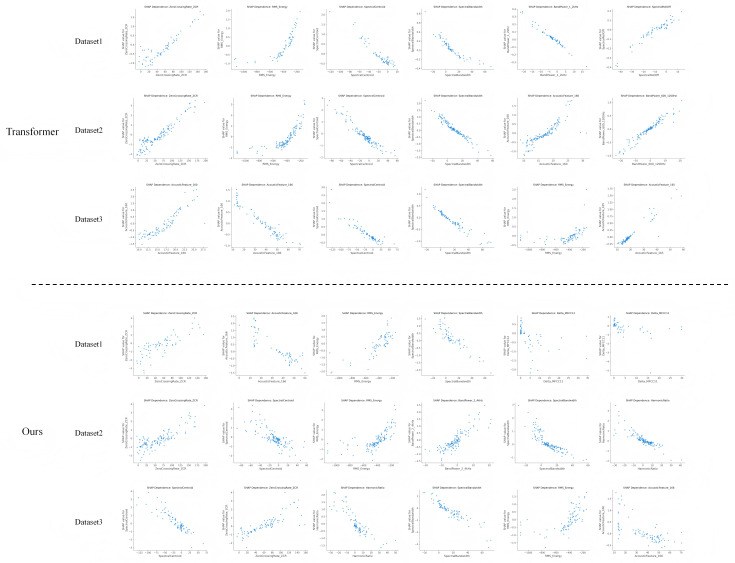
Dependency graph comparison between Transformer and the algorithm in this paper.

To further analyze the differences in feature-level decision logic and interpretability, this study employs the SHAP (SHapley Additive exPlanations) method to visualize and compare the feature dependency relationships between the baseline Transformer model and the proposed algorithm. By plotting the SHAP dependence diagrams of different models, the influence direction and strength of each acoustic feature on the prediction results can be intuitively observed, revealing how the medical guidance mechanism reshapes attention distribution and feature importance. This experiment aims to analyze the decision logic of the model in the time–frequency acoustic space from the perspective of feature contribution, thereby providing intuitive support for medical interpretability modeling. The experimental results are shown in Fig 9.

As shown in Fig 9, the proposed model exhibits a more stable and medically consistent feature response pattern in the SHAP dependency plots. Compared with the baseline Transformer, the feature dependency curves of the proposed model are smoother and display clearer monotonic relationships, indicating that the model responds to variations in acoustic feature values with greater consistency and interpretability. In particular, for key medical acoustic indicators such as *RMS Energy*, *Spectral Centroid*, and *Harmonic Ratio*, the model demonstrates positive correlation trends that align well with clinical observations, reflecting its heightened sensitivity to high-energy bursts and resonance-enhanced acoustic patterns in pertussis samples.

In contrast, the SHAP dependency distribution of the Transformer model appears more scattered, with several features exhibiting non-monotonic or even inverse fluctuations, suggesting that its attention mechanism, without medical constraints, is more prone to interference from redundant acoustic features. The incorporation of the MGIAM module introduces medically guided attention reallocation, enabling the model to capture acoustically and diagnostically meaningful feature variation patterns at the feature level. Consequently, the proposed approach achieves greater consistency and medical rationality in its predictive explanations. Overall, these results confirm that the proposed algorithm provides dual improvements in both interpretability of model decisions and stability of feature representations.

### 5.5 Model stability analysis

To verify the robustness and generalization ability of the proposed model in practical application scenarios, a model stability analysis experiment was further conducted. Specifically, Gaussian noise with varying amplitudes was artificially added to the input audio data to simulate real-world conditions such as recording device interference, background noise, and respiratory sound fluctuations, thereby examining the model’s ability to maintain performance under noise perturbations. This experiment aims to evaluate whether the proposed time–frequency fusion and medically guided mechanisms can enhance the model’s resistance to acoustic noise interference, providing a reliable foundation for the clinical applicability of medical audio recognition tasks. The experimental results are presented in Table 6.

**Table 6:** Model stability analysis under different noise intensities across three datasets (Mean ± Std).

Dataset 1					
Noise Intensity	Acc	Precision	Recall	F1-Score	AUC
0.3	0.914±0.010	0.926±0.011	0.893±0.012	0.909±0.010	0.979±0.008
0.2	0.925±0.009	0.936±0.010	0.906±0.010	0.921±0.009	0.986±0.007
0.1	0.933±0.008	0.944±0.009	0.913±0.009	0.928±0.008	0.991±0.006
0.0	0.938±0.008	0.956±0.010	0.935±0.009	0.946±0.008	0.994±0.006
**Dataset 2**					
0.3	0.873±0.011	0.924±0.012	0.816±0.012	0.866±0.011	0.956±0.008
0.2	0.884±0.010	0.935±0.011	0.829±0.011	0.878±0.010	0.964±0.007
0.1	0.893±0.009	0.942±0.010	0.837±0.010	0.886±0.009	0.969±0.006
0.0	0.900±0.008	0.947±0.010	0.843±0.009	0.892±0.008	0.984±0.006
**Dataset 3**					
0.3	0.945±0.008	0.933±0.010	0.956±0.009	0.944±0.008	0.987±0.005
0.2	0.954±0.007	0.943±0.009	0.965±0.008	0.953±0.007	0.992±0.005
0.1	0.960±0.007	0.949±0.008	0.971±0.007	0.959±0.007	0.994±0.004
0.0	0.965±0.006	0.953±0.008	0.976±0.007	0.964±0.006	0.996±0.004

As shown in Table 6, the proposed model maintains high stability and robustness under different noise intensities. As the noise amplitude increases from 0.0 to 0.3, although all evaluation metrics experience slight declines, the overall performance degradation remains minimal, indicating that the model can still effectively capture key acoustic features even in noisy environments. This stability benefits from the adaptive time–frequency fusion module, which dynamically models multi-scale acoustic information, as well as the medically guided attention mechanism, which adaptively suppresses noise within the feature space. Overall, these results demonstrate that the model maintains high classification accuracy and generalization capability under real-world complex acoustic conditions, providing a solid foundation for the practical deployment of medical audio recognition tasks.

### 5.6 Computational cost analysis

In this section, we analyze the computational cost of the proposed model and compare it with the training and inference costs of the baseline model under the same experimental settings to evaluate the efficiency and scalability of the method in real-world deployment scenarios. Specifically, we report and compare metrics such as training time, inference latency, and parameter size of different models to characterize the computational cost behind the performance improvement. The experimental results are shown in Table 7.

**Table 7:** Computational cost comparison between the Transformer baseline and the proposed model.

Method	Parameters	Training time	Test Time
Baseline	65.3M	3.7h	57ms (RTX 4090)
Ours	72.1M	4.2h	68ms (RTX 4090)

As shown in Table 7, the proposed model increases the parameter size only moderately compared with the Transformer baseline (72.1M vs. 65.3M, about a 10% increase), indicating that the architectural enhancements introduce limited additional model complexity. This relatively small growth in capacity leads to a proportional rise in training time (4.2h vs. 3.7h) and inference latency (68ms vs. 57ms on an RTX 4090), suggesting that the computational overhead remains controlled rather than exploding with model redesign. Overall, the results imply a favorable efficiency–performance trade-off: the proposed method achieves its gains with a modest extra compute budget, maintaining practical scalability for real-world deployment where both throughput and resource constraints matter.

## 6 Discussion

The results indicate that integrating adaptive time–frequency modeling with medically guided interpretable attention can provide a clinically meaningful balance between recognition accuracy and decision transparency for pertussis cough sound analysis. From a medical perspective, pertussis coughs exhibit non-stationary acoustic transitions and intermittent high-energy bursts that are easily confounded by coexisting respiratory conditions and recording variability, especially in pediatric settings where symptom presentation and sound quality can be inconsistent. By encouraging the model to focus on clinically relevant acoustic cues rather than spurious noise patterns, the proposed framework offers more inspectable evidence to support screening-oriented decision making, which is important for improving trust and potential usability in practical respiratory triage workflows. It should also be noted that SHAP and MGIAM may not always produce fully identical interpretations in all cases. SHAP mainly quantifies the contribution of input features to the final prediction outcome, whereas MGIAM emphasizes the time–frequency regions that the model preferentially attends to under medical guidance. Therefore, occasional local discrepancies between them should be understood as reflecting differences in interpretation granularity and explanation mechanism, rather than necessarily indicating inconsistency or unreliability in the model.

Despite these encouraging findings, there remains substantial work to bridge the gap between research evaluation and real-world clinical deployment. In future work, we will further investigate clinical-oriented validation with medical experts, including protocol-driven assessment of whether the highlighted time–frequency regions align with clinically recognized cough phases and acoustic indicators, and will explore prospective data collection under realistic hospital and community environments to evaluate generalization across devices and patient populations. In addition, although the current inference latency is acceptable under a high-performance computing environment, it is not yet sufficient to support stable real-time processing on a standard smartphone using only on-device hardware. In practical deployment, real-time screening would be more feasible when the mobile device is connected to a backend server equipped with high-performance accelerators. Therefore, further lightweight redesign, model compression, and hardware-aware optimization remain important directions for improving mobile accessibility in future work. Moreover, to facilitate mobile accessibility and real-time screening, we plan to optimize the computational footprint and inference latency of the model, building on recent efforts in CNN-based mobile optimization for lung sound classification [39], and conduct targeted clinical studies to assess feasibility, safety, and workflow integration in clinical settings.

## 7 Conclusion

This study addresses the challenges of complex feature distributions and insufficient interpretability in pertussis cough sound recognition by proposing a deep learning framework based on adaptive time–frequency fusion and medically guided attention mechanisms. The proposed method establishes a dynamic feature fusion structure across temporal and spectral dimensions while incorporating medical acoustic priors into attention allocation, thereby achieving fine-grained modeling of pertussis acoustic patterns and visual interpretability with medical significance. Experimental results demonstrate that the model exhibits superior recognition performance and robust generalization across multiple datasets while significantly enhancing the interpretability of its outputs, providing a feasible pathway for the trustworthy application of medical AI systems in respiratory disease screening. Moreover, the deformable multi-head attention mechanism strengthens the model’s ability to dynamically locate key cough segments, and the medically guided attention mapping ensures consistency between the model’s decision-making process and acoustic feature semantics, achieving a balance between performance and interpretability. The significance of this research lies not only in improving the automation accuracy of pertussis recognition but also in validating the feasibility of integrating deep models with medical knowledge, offering a new paradigm for small-sample medical audio recognition.

Future work will proceed in three directions: first, expanding the dataset scale and exploring cross-region and cross-device model transferability; second, integrating federated learning and privacy-preserving computation frameworks to construct a distributed medical acoustic recognition platform; and third, investigating multi-disease cough sound joint modeling and multimodal diagnostic mechanisms to enable a more comprehensive intelligent analysis of respiratory systems.
